# A non-linear association of low-density lipoprotein cholesterol with all-cause and cardiovascular mortality among patients with hypertension

**DOI:** 10.3389/fcvm.2024.1469848

**Published:** 2024-12-23

**Authors:** Guoliang Liang, Wenhao Zhang, Xinxin Gu, Qiong Zhang, Ankang Liu, Xinran Qing, Jiangwei Ma

**Affiliations:** ^1^ Department of Cardiovascular Medicine, Fengxian District Central Hospital, Shanghai, China; ^2^ Medical School of Anhui University of Science and Technology, Huainan, China

**Keywords:** low-density lipoprotein cholesterol, all-cause mortality, cardiovascular mortality, hypertension, NHANES

## Abstract

**Background:**

Although a few studies have examined the correlation between low-density lipoprotein cholesterol (LDL-C) and mortality, no study has explored these associations in hypertensive populations. This study aims to investigate the relationship between low-density lipoprotein cholesterol and cardiovascular and all-cause mortality in adults with hypertension.

**Methods:**

Hypertensive participants aged ≥18 years from the National Health and Nutrition Examination Survey 1999–2018 with blood lipid testing data and complete follow-up data until 31 December 2019 were enrolled in the analysis. Univariate and multivariate Cox regression were conducted for the calculation of hazard ratios and 95% confidence intervals. A restricted cubic spline curve was performed to visually represent the relationship between LDL-C and mortality. Kaplan–Meier survival analysis and stratification analysis were also carried out.

**Results:**

We finally analysed a cohort of 9,635 participants (49.6% male, mean age of 59.4 years). After a median follow-up of 98 months, there were 2,283 (23.7%) instances of all-cause fatalities, with 758 (7.9%) cases attributed to cardiovascular disease. Multivariate Cox regression analysis showed that lower levels of LDL-C were associated with a higher risk of all-cause and cardiovascular mortality; the LDL-C group’s lowest level (<2.198 mmol/L) still showed a 19.6% increased risk of all-cause mortality (*p* = 0.0068) in the model that was completely adjusted. Both all-cause mortality and cardiovascular mortality showed a non-linear association with LDL-C concentration in restricted cubic spline regression analysis.

**Conclusions:**

In individuals with hypertension, LDL-C was linked to cardiovascular and all-cause mortality. It was further demonstrated that this relationship was non-linear.

## 1 Introduction

In recent decades, low-density lipoprotein cholesterol (LDL-C) has been recognised as a risk factor for atherosclerosis and cardiovascular disease (1–3). There has been evidence from several studies in various populations throughout the world that higher LDL-C levels are associated with mortality due to all-cause and cause-specific (cardiovascular disease, stroke, etc.) mortality (4–8). A large number of randomised controlled trials also demonstrated that lipid-lowering drugs were associated with a lower risk of cardiovascular events and mortality associated with atherosclerosis (9–12). However, with further research, conflicting results were revealed regarding LDL-C levels and mortality risk. Some studies found deaths from all causes were negatively correlated with LDL-C levels (13, 14) and some showed no association (15, 16). A recent extensive prospective cohort study in Denmark revealed a U-shaped correlation between different LDL-C levels and death (17). The relationship between concentrations of LDL-C and mortality remains unclear.

Moreover, we noticed that studies are usually conducted in the general population, older population, or diabetic population; to date, no study has focused on patients with hypertension. Globally, the hypertensive population has reached a staggering number of 874 million, and approximately one in four adults has hypertension (18). Previous studies have shown that hypertension is associated with dyslipidaemia, and the LDL-C may be a modifiable risk factor for hypertension on its own (19, 20). A study by Bønaa et al. showed a positive correlation between blood pressure and lipid levels (20). Hence, having such a large population with hypertension, providing some guidelines for risk hierarchy management of those patients and investigating the associations between LDL-C levels and all-cause and cardiovascular mortality may be helpful.

## 2 Methods

### 2.1 Data source and study population

In this cohort study, all data were obtained from the National Health and Nutrition Examination Survey (NHANES, https://www.cdc.gov/nchs/nhanes/index.htm). NHANES is a major programme of the National Center for Health Statistics (NCHS), which is part of the Centers for Disease Control and Prevention (CDC). The programme aims to assess the health and nutritional status of adults and children in the United States (21). Since 1999, health data gathered by interviews, physical exams, and laboratory testing from representative American population samples would be published on their official website every 2 years (22) and ongoing follow-up mortality data would also be posted in the National Death Index death certificate records (www.cdc.gov/nchs/data-linkage/mortality-public.htm).

**Figure 1:**
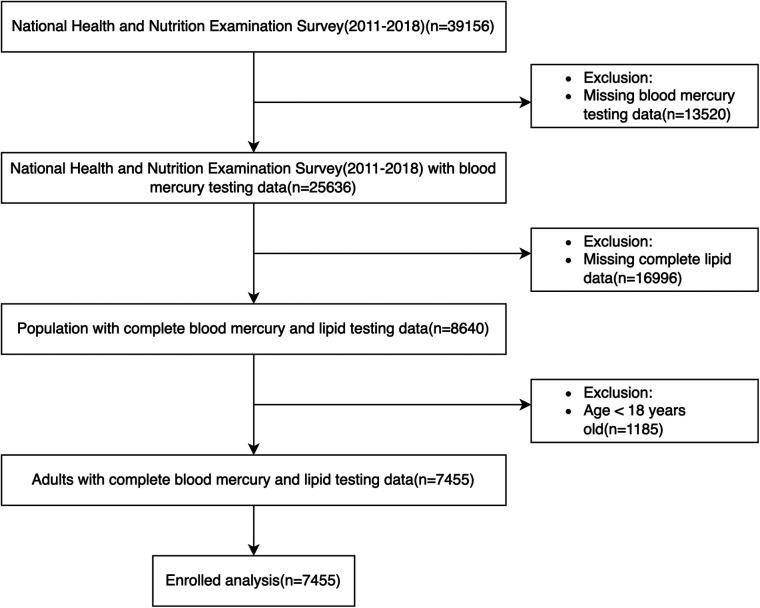
Flow chart of participants selected from NHANES 1999–2018.

In our analysis, we collected 11 cycles of datasets (NHANES 1999–2018, each cycle is 2 years) and then extracted demographic data, blood pressure, and body mass index (BMI) data from examinations, lipid testing data from laboratories, questionnaire data about smoking status, and use of antihypertensive and lipid-lowering drugs. Participants aged under 18 years, those with missing blood lipid and follow-up data, missing body mass index and smoking data, or those without hypertension at baseline were excluded from the study. After the application of the above criteria for exclusion, 9,635 individuals were ultimately included for analysis (Figure 1). Participants’ survival status and death details were tracked through 31 December 2019. The Centers for Disease Control and Prevention's Institutional Review Board gave its approval to the NHANES research methodology. Every participant provided their consent before participating, and all of the methods for the survey were conducted in accordance with the relevant rules and regulations (https://www.cdc.gov/nchs/nhanes/about_nhanes.htm).

### 2.2 Data collection and potential confounding variables

Demographic information (age, gender, race, marital status, and education level) was recorded at the beginning of every cycle of the survey as questionnaire data. We extracted it from the 11 cycles of datasets mentioned above and converted race, marital status, and level of education to binary categorical variables. Race was categorised as White (Mexican American, Other Hispanic, Non-Hispanic White)/non-White (Non-Hispanic Black, Other Race), marital status was categorised as Married/Other (Widowed, Divorced, Separated, Refused, etc.), and level of education was categorised as Less than high school (Less Than 9th Grade, 9–11th Grade, Refused, Don’t Know) or High school or above (High School Grad/GED or Equivalent, Some College or AA degree, College Graduate or above).

Blood pressure and body measure data were collected and stored in the examination data module. We extracted systolic blood pressure, diastolic blood pressure, and BMI data. Referring to the American Heart Association Blood Pressure Guidelines 2018, we defined hypertension as systolic blood pressure ≥140 mmHg and/or diastolic blood pressure ≥90 mmHg or self-reported hypertension history and use of antihypertensive medication (1, 23). According to the NHANES component description, BMI was calculated using weight (kg) divided by the square of height (m^2^).

Cholesterol measurement data were stored in the module of laboratory data. We extracted total cholesterol (TC), triglyceride (TG), low-density lipoprotein cholesterol (LDL-C), and high-density lipoprotein cholesterol (HDL-C) concentration (mmol/L) data for our study. According to NHANES instructions, all measurements were taken in the morning on an empty stomach (fasting for at least 8 h).

Smoking status questionnaire data were also extracted. We defined the answer of SMQ020 (Have you/Has SP smoked at least 100 cigarettes in your/his/her entire life?): Yes means smoking; No/Refused/Don't know/Missing means no smoking. As to medication history, the questionnaire data of BPQ050A (Are you/Is SP now taking prescribed medicine for hypertension?) and BPQ100D [Are you/Is SP now following taking prescribed medicine to lower (your/his/her) blood cholesterol?] were selected to define the following: answer Yes means yes; No/Refused/Don't know/Missing means no.

We included demographic information (age, gender, race, marital status, education level), body measure data (body mass index, blood pressure), and personal lifestyle habits (smoking status, medication history) as potential confounding variables. These potential confounders were included in this analysis based on previous literature (7, 8, 15) and data from NHANES.

### 2.3 Outcomes and follow-up

All-cause mortality and cardiovascular mortality were selected as our study outcomes. All-cause mortality is defined as death from any cause, including diseases of the heart, malignant neoplasms, chronic lower respiratory diseases, accidents, cerebrovascular diseases, Alzheimer's disease, diabetes mellitus, influenza and pneumonia, nephritis, and all other causes.

Cardiovascular mortality was estimated using The International Classification of Diseases, 10th Revision (ICD-10), and codes (I00–I09, I11, I13, I20–I51, and I60–I69) were used to define cardiovascular deaths. The mortality data of NHANES 1999–2018 were linked to mortality data from the National Death Index death certificate records until 31 December 2019.

All participants enrolled in this study had complete follow-up data. When death occurred, causes of death were recorded.

### 2.4 Statistical analysis

To describe the differences in mortality risks between different LDL-C concentration levels, we referred to some previous literature (23) using commonly used statistical grouping methods and divided LDL-C levels into five groups based on quintiles (Q1: <20th percentile, Q2: ≤20–40th percentile, Q3: ≤40–60th percentile, Q4: ≤60–80th percentile, Q5: ≥80th percentile). To facilitate comparison and illustrate the mortality risk relationship between groups of LDL-C levels, the LDL-C level of Q3 (2.689–3.155 mmol/L) was selected as a reference to study the relationship between LDL-C and all-cause and cardiovascular mortality.

In this study, continuous variables were described by means ± standard deviations (SD) and compared using an analysis of variance (ANOVA). We compared categorical variables using the chi-square test, expressing them as numbers (*n*) and percentages (%). We used univariate Cox regression to identify potential risk factors that may affect all-cause mortality and cardiovascular mortality, and the results are represented as hazard ratios (HRs) with 95% confidence intervals (CIs). In this session, statistically significant categorical variables and continuous variables previously shown to be associated with all-cause and cardiovascular mortality will be included in multivariate Cox regression analysis. An analysis of multivariate Cox regression models was carried out to determine if LDL-C levels are associated with mortality due to all causes and cardiovascular disease. Three models were constructed: model I is a crude model and adjusts for none; model II adjusts for age, gender, and race; and model III is a comprehensive model that includes adjustments for smoking, systolic blood pressure, diastolic blood pressure, and medication use (antihypertensive medicines, lipid-lowering medicines) beyond those included in model II. To control for confounding bias and to quadratically validate the accuracy of the statistical inferences we made, we used inverse probability-weighted Cox regression analysis. A restricted cubic spline (RCS) curve was used to analyse and visualise the relationship between LDL-C concentration and mortality on a continuous scale, which is based on multivariate-adjusted Cox regression. The Kaplan–Meier curve for survival analysis was carried out to show how survival varies between different level groups of LDL-C. Finally, we also conducted a stratification analysis to identify the subgroup that shows a significant connection between LDL-C level and all-cause and cardiovascular death, including age, gender, race, marital status, level of education, smoking, body mass index, and medicine use. The statistical significance level was determined by *p* < 0.05 on two sides. All statistical analysis were performed using R version 4.3.1 (R Foundation for Statistical Computing, Vienna, Austria, https://www.r-project.org/).

## 3 Results

### 3.1 Characteristics of the study population at different levels of LDL

Table 1 shows the distribution of baseline characteristics of research participants based on various levels of LDL-C concentrations. A total of 9,635 participants were included in our analysis, of which 49.6% were men and 50.4% were women (mean age 59.4 ± 15.6 years). Most of them were White (68.2%) and received a high school or above education (85.7%). After a median follow-up of 98 months, there were 2,283 instances of all-cause fatalities, with 758 cases attributed to cardiovascular disease. Compared with the higher groups (Q4 and Q5), the lower groups (Q1 and Q2) were more likely to be older, male, smoker, and were more likely to take antihypertensive drugs and lipid-lowering drugs. Among various LDL-C classification levels, except for marital status, education level, and body mass index, all other social demographic and health-related disease factors show statistical significance at baseline (*p* < 0.05). Table 2 demonstrates the baseline after adjusting for the confounders of age, gender, race, marital status, smoking status, level of education, and use of lipid-lowering and antihypertensive medication using an inverse probability weighting approach.

**Table 1:** LDL-C level quintile-based baseline characteristics of study cohort.

Characteristics	Total	Quintiles of the LDL-C (mmol/L)					*p*
		Q1 (<2.198)	Q2 (2.198–2.689)	Q3 (2.689–3.155)	Q4 (3.155–3.75)	Q5 (≥3.75)	
Number	9,635	1,921	1,901	1,913	1,947	1,953	
Age (years)	59.4 ± 15.6	62.2 ± 16.0	59.5 ± 16.2	58.7 ± 15.3	58.5 ± 15.4	58.3 ± 14.6	<0.001
Gender, *n* (%)							<0.001
Male	4,780 (49.6)	1,037 (54.0)	955 (50.2)	935 (48.9)	949 (48.7)	904 (46.3)	
Female	4,855 (50.4)	884 (46.0)	946 (49.8)	978 (51.1)	998 (51.3)	1,049 (53.7)	
Race, *n* (%)							0.005
White	6,570 (68.2)	1,265 (65.9)	1,277 (67.2)	1,332 (69.6)	1,380 (70.9)	1,316 (67.4)	
Non-White	3,065 (31.8)	656 (34.1)	624 (32.8)	581 (30.4)	567 (29.1)	637 (32.6)	
Marital status, *n* (%)							0.112
Married	5,211 (54.1)	1,036 (53.9)	1,058 (55.7)	991 (51.8)	1,079 (55.4)	1,047 (53.6)	
Other	4,424 (45.9)	885 (46.1)	843 (44.3)	922 (48.2)	868 (44.6)	906 (46.4)	
Education level, *n* (%)							0.800
Less than high school	1,381 (14.3)	280 (14.6)	271 (14.3)	269 (14.1)	267 (13.7)	294 (15.1)	
High school or above	8,254 (85.7)	1,641 (85.4)	1,630 (85.7)	1,644 (85.9)	1,680 (86.3)	1,659 (84.9)	
Smoking, *n* (%)							<0.001
No	4,865 (50.5)	903 (47.0)	912 (48.0)	979 (51.2)	1,036 (53.2)	1,035 (53.0)	
Yes	4,770 (49.5)	1,018 (53.0)	989 (52.0)	934 (48.8)	911 (46.8)	918 (47.0)	
Body mass index (kg/m^2^)	30.4 ± 7.15	30.5 ± 7.53	30.4 ± 7.17	30.6 ± 7.57	30.5 ± 6.87	30.2 ± 6.59	0.523
Systolic blood pressure (mmHg)	137 ± 20.2	134 ± 20.1	136 ± 20.1	136 ± 20.6	137 ± 19.1	140 ± 20.9	<0.001
Diastolic blood pressure (mmHg)	73.0 ± 13.8	69.1 ± 13.6	72.3 ± 13.5	73.0 ± 13.7	74.3 ± 13.4	76.4 ± 13.5	<0.001
Total cholesterol (mmol/L)	5.06 ± 1.09	3.75 ± 0.569	4.49 ± 0.453	4.98 ± 0.441	5.52 ± 0.448	6.53 ± 0.751	<0.001
HDL cholesterol (mmol/L)	1.39 ± 0.425	1.35 ± 0.464	1.40 ± 0.431	1.40 ± 0.434	1.38 ± 0.399	1.40 ± 0.392	<0.001
Triglycerides (mmol/L)	1.50 ± 0.789	1.40 ± 0.863	1.44 ± 0.798	1.46 ± 0.754	1.56 ± 0.756	1.64 ± 0.748	<0.001
LDL cholesterol (mmol/L)	2.99 ± 0.950	1.75 ± 0.328	2.44 ± 0.140	2.91 ± 0.135	3.43 ± 0.170	4.38 ± 0.599	<0.001
Antihypertensive drugs, *n* (%)							<0.001
No	4,051 (42.0)	531 (27.6)	712 (37.5)	837 (43.8)	932 (47.9)	1,039 (53.2)	
Yes	5,584 (58.0)	1,390 (72.4)	1,189 (62.5)	1,076 (56.2)	1,015 (52.1)	914 (46.8)	
Lipid-lowering drugs, *n* (%)							<0.001
No	6,776 (70.3)	995 (51.8)	1,180 (62.1)	1,419 (74.2)	1,568 (80.5)	1,614 (82.6)	
Yes	2,859 (29.7)	926 (48.2)	721 (37.9)	494 (25.8)	379 (19.5)	339 (17.4)	
Outcomes, *n* (%)							
All-cause mortality							<0.001
No	7,352 (76.3)	1,409 (73.3)	1,421 (74.8)	1,465 (76.6)	1,528 (78.5)	1,529 (78.3)	
Yes	2,283 (23.7)	512 (26.7)	480 (25.2)	448 (23.4)	419 (21.5)	424 (21.7)	
Cardiovascular mortality							<0.001
No	8,877 (92.1)	1,739 (90.5)	1,746 (91.8)	1,768 (92.4)	1,827 (93.8)	1,797 (92.0)	
Yes	758 (7.9)	182 (9.5)	155 (8.2)	145 (7.6)	120 (6.2)	156 (8.0)	

Q, quintiles; *n*, number; HDL, high-density lipoprotein; LDL, low-density lipoprotein.

Values are mean ± standard deviation for continuous variables or *n* (%) for categorical variables.

**Table 2:** Baseline characteristics based on quintiles of LDL-C levels after IPTW.

Characteristics	Total	Quintiles of the LDL-C (mmol/L)					*p*
		Q1 (<2.198)	Q2 (2.198–2.689)	Q3 (2.689–3.155)	Q4 (3.155–3.75)	Q5 (≥3.75)	
Number	9,637.26	1,913.46	1,932.34	1,899.03	1,953.4	1,939.03	
Age (years)	58.9 ± 15.70	59.32 ± 14.94	58.40 ± 17.39	59.25 ± 16.35	59.23 ± 1 5.07	58.63 ± 1 4.57	0.348
Gender, *n* (%)							0.596
Male	4,702.22 (48.79)	945.37 (49.41)	913.39 (47.27)	942.06 (49.61)	965.50 (49.43)	935.90 (48.27)	
Female	4,935.04 (51.21)	968.09 (50.59)	1,018.95 (52.73)	956.97 (50.39)	987.90 (50.57)	1,003.13 (51.73)	
Race, *n* (%)							0.804
White	6,524.81 (67.70)	1,302.83 (68.09)	1,283.49 (66.42)	1,291.91 (68.03)	1,326.41 (67.90)	1,320.17 (68.08)	
Non-White	3,112.45 (32.30)	610.63 (31.91)	648.85 (33.58)	607.12 (31.97)	626.99 (32.10)	618.86 (31.92)	
Marital status, *n* (%)							0.969
Married	5,163.09 (53.97)	1,033.22 (54.00)	1,023.05 (52.94)	1,022.33 (53.83)	1,051.20 (53.81)	1,033.29 (53.29)	
Other	4,474.17 (46.43)	880.24 (46.00)	909.29 (47.06)	876.70 (46.17)	902.20 (46.19)	905.74 (46.71)	
Education level, *n* (%)							0.992
Less than high school	1,356.79 (14.08)	272.94 (14.26)	265.74 (13.75)	268.21 (14.12)	278.67 (14.27)	271.25 (13.99)	
High school or above	8,280.47 (85.92)	1,640.52 (85.74)	1,666.60 (86.25)	1,630.82 (85.88)	1,674.73 (85.73)	1,667.78 (86.01)	
Smoking, *n* (%)							0.997
No	4,855.59 (50.38)	969.11 (50.65)	976.82 (50.55)	955.83 (50.33)	977.32 (50.03)	976.51 (50.36)	
Yes	4,781.67 (49.62)	944.35 (49.35)	955.52 (49.45)	943.20 (49.67)	976.08 (49.97)	962.52 (49.64)	
Body mass index (kg/m^2^)	30.45 ± 7.27	30.54 ± 7.51	30.56 ± 8.13	30.26 ± 7.19	30.53 ± 6.87	30.37 ± 6.56	0.703
Systolic blood pressure (mmHg)	136.64 ± 20.26	136.65 ± 20.93	136.40 ± 20.28	136.69 ± 20.43	136.59 ± 19.29	136.85 ± 20.37	0.982
Diastolic blood pressure (mmHg)	73.13 ± 13.89	73.05 ± 14.01	73.22 ± 13.73	73.10 ± 13.78	72.92 ± 14.00	73.35 ± 13.96	0.936
Total cholesterol (mmol/L)	5.07 ± 1.08	4.98 ± 0.44	3.80 ± 0.59	4.50 ± 0.46	5.52 ± 0.45	6.52 ± 0.75	<0.001
HDL cholesterol (mmol/L)	1.39 ± 0.44	1.41 ± 0.43	1.39 ± 0.50	1.41 ± 0.44	1.37 ± 0.40	1.40 ± 0.40	0.137
Triglycerides (mmol/L)	1.50 ± 0.80	1.46 ± 0.75	1.40 ± 0.89	1.43 ± 0.80	1.57 ± 0.76	1.66 ± 0.76	<0.001
Antihypertensive drugs, *n* (%)							0.915
No	4,074.57 (42.28)	806.23 (42.13)	830.06 (42.96)	795.69 (41.90)	812.65 (41.60)	829.94 (42.80)	
Yes	5,562.69 (57.72)	1,107.23 (57.87)	1,102.82 (57.04)	1,103.34 (58.10)	1,140.75 (58.40)	1,109.09 (57.20)	
Lipid-lowering drugs, *n* (%)							0.96
No	6,786.65 (70.42)	1,345.46 (70.32)	1,365.50 (70.67)	1,331.87 (70.13)	1,366.39 (69.95)	1,377.42 (71.04)	
Yes	2,850.61 (29.58)	568.00 (29.68)	566.84 (29.33)	567.16 (29.87)	587.01 (34.05)	561.61 (28.96)	
Outcomes, *n* (%)							
All-cause mortality							0.047
No	7,363.18 (76.40)	1,462.25 (76.42)	1,469.32 (76.04)	1,403.72 (73.92)	1,515.47 (77.58)	1,512.43 (78.00)	
Yes	2,274.08 (23.60)	451.21 (23.58)	463.02 (23.96)	495.31 (26.08)	437.93 (22.42)	426.60 (22.00)	
Cardiovascular mortality							0.29
No	8,891.98 (92.27)	1,767.84 (92.39)	1,778.44 (92.04)	1,740.86 (91.67)	1,825.6 (93.74)	1,779.59 (91.78)	
Yes	745.28 (7.73)	145.62 (7.61)	153.90 (7.96)	158.17 (8.33)	128.14 (6.56)	159.44 (8.22)	

IPTW, inverse probability of treatment weighting; Q, quintiles; *n*, number; HDL, high-density lipoprotein.

Values are mean ± standard deviation for continuous variables or *n* (%) for categorical variables.

### 3.2 HRs of LDL-C levels for all-cause and cardiovascular mortality

To identify potential risk factors that affect all-cause and cardiovascular mortality, we first used a univariate Cox regression analysis to explore how the categorical variables included in this analysis affected all-cause mortality and cardiovascular mortality. As the results show in Table 3, when the medium level of LDL-C (2.689–3.155 mmol/L) was selected as a reference group, the lower level of the LDL-C group had a higher risk of all-cause and cardiovascular death. The lowest level of the LDL-C group (<2.198 mmol/L) had a 1.459 (95% CI 1.285–1.657) times higher risk of all-cause death and a 1.609 (95% CI 1.293–2.002) times higher risk of cardiovascular death than the reference group. The univariate analysis also revealed that participants who were men, non-White, smokers, had a lower level of education, with marital status of “other,” using antihypertensive drugs, and now taking lipid-lowering drugs had a higher risk of all-cause and cardiovascular death (all *p* < 0.05).

**Table 3:** Result of univariate analysis.

Characteristics	All-cause mortality		*p*	Cardiovascular mortality		*p*
	Events/numbers	HR (95% CI)		Events/numbers	HR (95% CI)	
LDL-C						
<2.198	512/1,921	1.459 (1.285–1.657)	<0.001	182/1,921	1.609 (1.293–2.002)	<0.001
2.198–2.689	480/1,901	1.218 (1.070–1.385)	0.0,027	155/1,901	1.217 (0.970–1.527)	0.089
2.689–3.155	448/1,913	1 (Ref)		145/1,913	1 (Ref)	
3.155–3.75	419/1,947	0.874 (0.765–0.998)	0.0,466	120/1,947	0.772 (0.606–0.984)	0.036
≥3.75	424/1,953	0.847 (0.742–0.967)	0.0,143	156/1,953	0.962 (0.767–1.206)	0.734
Gender						
Male	1,236/4,780	1 (Ref)		420/4,780	1 (Ref)	
Female	1,047/4,855	0.784 (0.722–0.851)	<0.001	338/4,855	0.744 (0.645–0.859)	<0.001
Race						
White	1,775/6,570	0.728 (0.659–0.803)	<0.001	575/6,570	0.811 (0.686–0.958)	0.014
Non-White	508/3,065	1 (Ref)		183/3,065	1 (Ref)	
Marital status						
Married	1,105/5,211	1 (Ref)		351/5,211	1 (Ref)	
Other	1,178/4,424	1.400 (1.290–1.520)	<0.001	407/4,424	1.520 (1.320–1.760)	<0.001
Education level						
Less than high school	485/1,381	1 (Ref)	<0.001	157/1,381	1 (Ref)	
High school or above	1,798/8,254	0.663 (0.600–0.733)		601/8,254	0.686 (0.576–0.818)	<0.001
Smoking						
No	937/4,865	0.651 (0.599–0.707)	<0.001	342/4,865	0.769 (0.667–0.887)	<0.001
Yes	1,346/4,770	1 (Ref)		416/4,770	1 (Ref)	
Antihypertensive drugs						
No	1,489/4,051	0.597 (0.547–0.651)	<0.001	238/4,051	0.510 (0.437–0.595)	<0.001
Yes	794/5,584	1 (Ref)		520/5,584	1 (Ref)	
Lipid-lowering drugs						
No	1,571/6,776	0.744 (0.681–0.814)	<0.001	500/6,776	0.650 (0.559–0.756)	<0.001
Yes	712/2,859	1 (Ref)		258/2,859	1 (Ref)	

HR, hazard ratio; CI, confidence interval; Ref, reference.

Table 4 shows the results of the multivariate Cox regression analysis of different LDL-C levels with all-cause and cardiovascular mortality. We provided three kinds of models. Model I was a crude model and adjusted for none. The results show that the lower level of LDL-C groups had a higher risk of all-cause and cardiovascular mortality. After adjusting for age, gender, and race, model II also found a higher risk of all-cause and cardiovascular mortality in lower levels of LDL-C groups. Model III was a fully adjusted model that adjusted for age, gender, race, smoking, marital status, level of education, body mass index, systolic blood pressure, diastolic blood pressure, and medicine use (antihypertensive drugs, lipid-lowering drugs). In this model, the lowest level of LDL-C group (<2.198 mmol/L) still showed a 19.6% increased risk of all-cause death (*p* = 0.0068), and the second lowest group (2.198–2.689 mmol/L) showed a 1.184 (95% CI 1.040–1.358) times higher risk of all-cause death than the reference group. However, differences in the risk of cardiovascular death were not so significant in the fully adjusted model.

**Table 4:** Result of multivariate Cox regression analysis.

	Model I	Model II	Model III
	HR (95% CI), *p*-value	HR (95% CI), *p*-value	HR (95% CI), *p*-value
All-cause mortality			
LDL-C levels			
<2.198	1.459 (1.285–1.657), <0.001	1.170 (1.029–1.329), 0.0160	1.196 (1.051–1.361), 0.0068
2.198–2.689	1.218 (1.070–1.385), 0.0027	1.166 (1.025–1.327), 0.0190	1.184 (1.040–1.348), 0.0108
2.689–3.155	1 (Ref)	1 (Ref)	1 (Ref)
3.155–3.75	0.874 (0.765–0.998), 0.0466	0.861 (0.753–0.983), 0.0270	0.852 (0.745–0.973), 0.0186
≥3.75	0.847 (0.742–0.967), 0.0143	0.898 (0.786–1.026), 0.1130	0.879 (0.769–1.006), 0.0611
Cardiovascular mortality			
LDL-C levels			
<2.198	1.609 (1.293–2.002), <0.001	1.242 (0.997–1.547), 0.0537	1.234 (0.987–1.542), 0.0647
2.198–2.689	1.217 (0.970–1.527), 0.0890	1.164 (0.927–1.460), 0.2265	1.153 (0.917–1.448), 0.2230
2.689–3.155	1 (Ref)	1 (Ref)	1 (Ref)
3.155–3.75	0.772 (0.606–0.984), 0.0360	0.761 (0.597–0.969), 0.0269	0.761 (0.597–0.970), 0.0272
≥3.75	0.962 (0.767–1.206), 0.7340	1.026 (0.818–1.288), 0.8219	1.012 (0.804–1.272), 0.9214

HR, hazard ratio; CI, confidence interval; Ref, reference.

Model I adjust for none. Model II adjust for age, gender, and race. Model III adjust for age, gender, race, marital status, education level, smoking, body mass index, systolic blood pressure, diastolic blood pressure, and medication use (antihypertensive and lipid-lowering medication).

Table 5 displays the results of the Cox regression analysis based on data from Table 1, which was adjusted by inverse probability of treatment weighting. Compared to the group with the lowest LDL-C levels, the risk of both all-cause and cardiovascular death was reduced in the group with higher LDL levels, which is consistent with our stepwise Cox regression results.

**Table 5:** Cox regression analysis based on data after IPTW.

Characteristics	All-cause mortality		Cardiovascular mortality	
	HR (95% CI)	*p*	HR (95% CI)	*p*
LDL-C				
<2.198	1 (Ref)		1 (Ref)	
2.198–2.689	1.036 (0.903–1.189)	0.6141	0.996 (0.791–1.250)	0.9727
2.689–3.155	0.847 (0.737–0.972)	0.0185	0.822 (0.652–1.040)	0.0971
3.155–3.75	0.777 (0.672–0.898)	<0.001	0.683 (0.530–0.880)	0.0031
≥3.75	0.739 (0.639–0.856)	<0.001	0.831 (0.654–1.060)	0.1311

IPTW, inverse probability of treatment weighting; HR, hazard ratio; CI, confidence interval; Ref, reference.

### 3.3 Association between LDL-C concentration and all-cause and cardiovascular mortality

**Figure 2:**
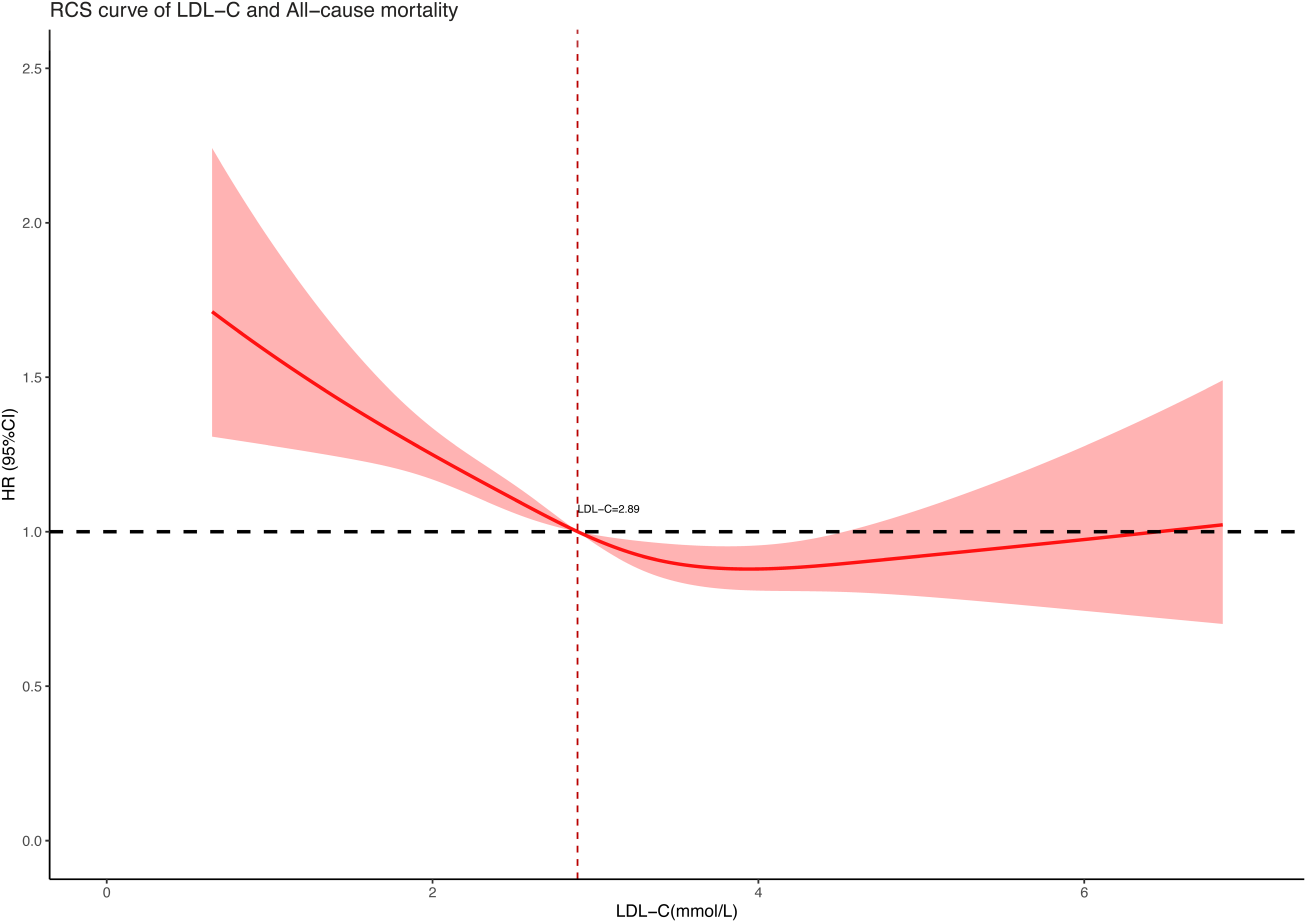
Restricted cubic spline curve of LDL-C concentration (mmol/L) and all-cause mortality.

**Figure 3:**
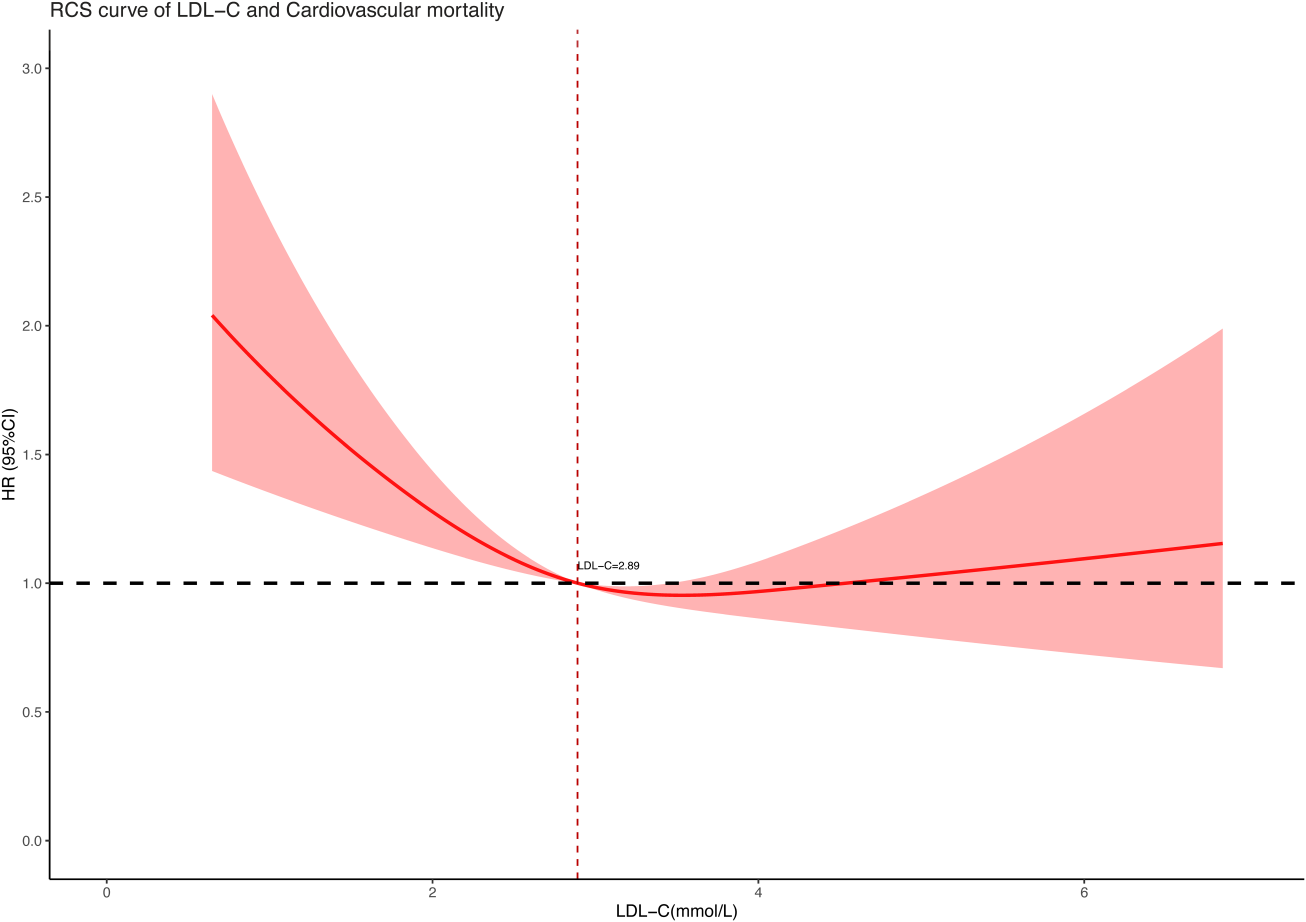
Restricted cubic spline curve of LDL-C concentration (mmol/L) and cardiovascular mortality.

To further explore the association between LDL-C concentration (as a continuous variable) and all-cause and cardiovascular mortality, we performed restricted cubic spline regression analysis on our included data and used an RCS curve to visually show the result. The analysis models were based on multivariate-adjusted Cox regression and fully adjusted for confounders. As shown in the results, both all-cause mortality (Figure 2) and cardiovascular mortality (Figure 3) had a non-linear association with LDL-C concentration. When LDL-C concentrations were below 2.89 mmol/L, both the risk of all-cause death and cardiovascular death became higher as the LDL-C concentration decreased. Risks of all-cause and cardiovascular death tended to further decrease and then increase when the blood LDL-C concentration was above 2.89 mmol/L. On the contrary, the risk of cardiovascular death seemed to increase at a lower LDL-C concentration than all-cause death. The red shaded area represents the 95% CI of the curve.

### 3.4 Survival analysis and stratification analysis

**Figure 4:**
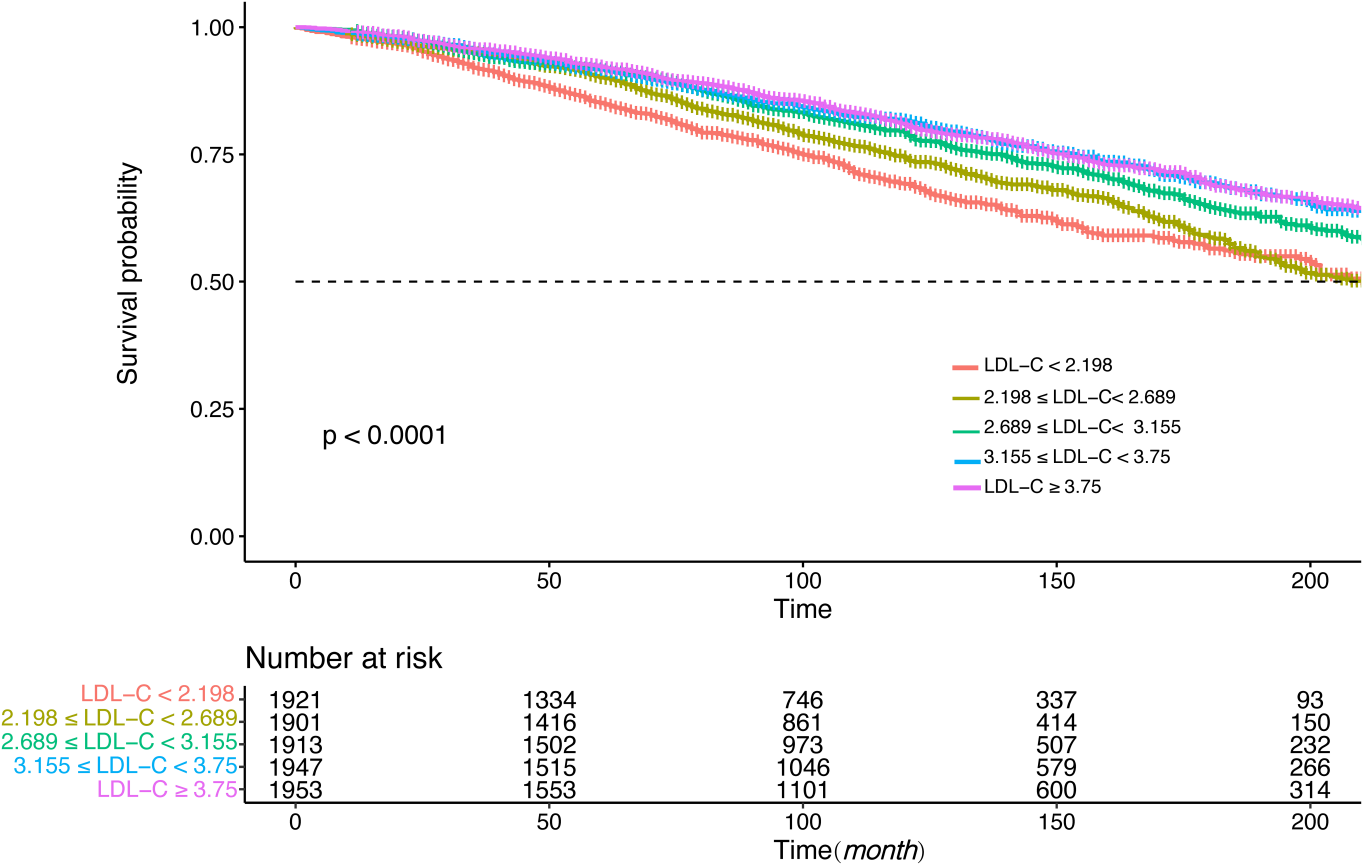
Kaplan–Meier curve of all-cause mortality for different levels of LDL-C concentration.

**Figure 5:**
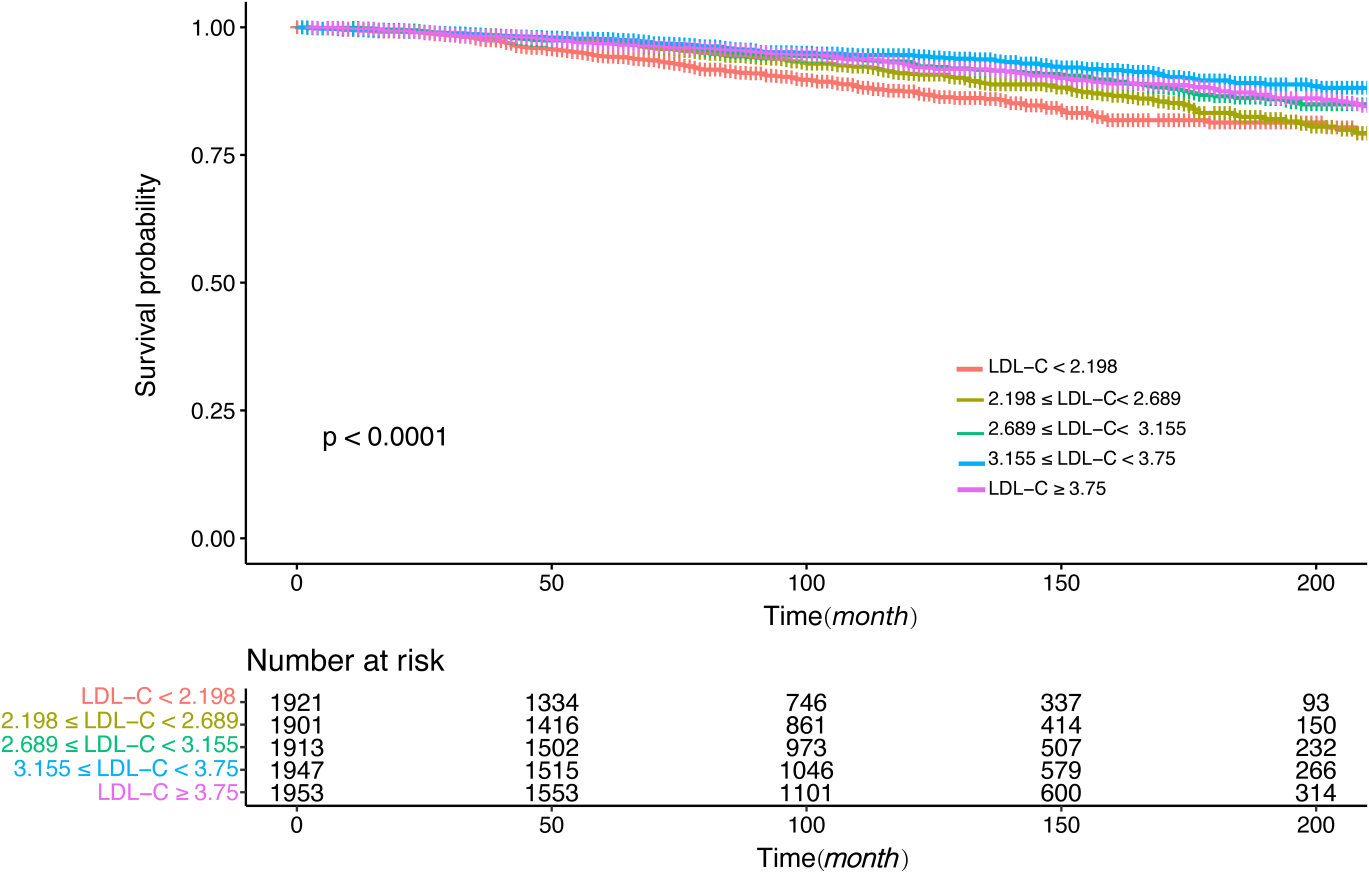
Kaplan–Meier curve of cardiovascular mortality for different levels of LDL-C concentration.

**Figure 6:**
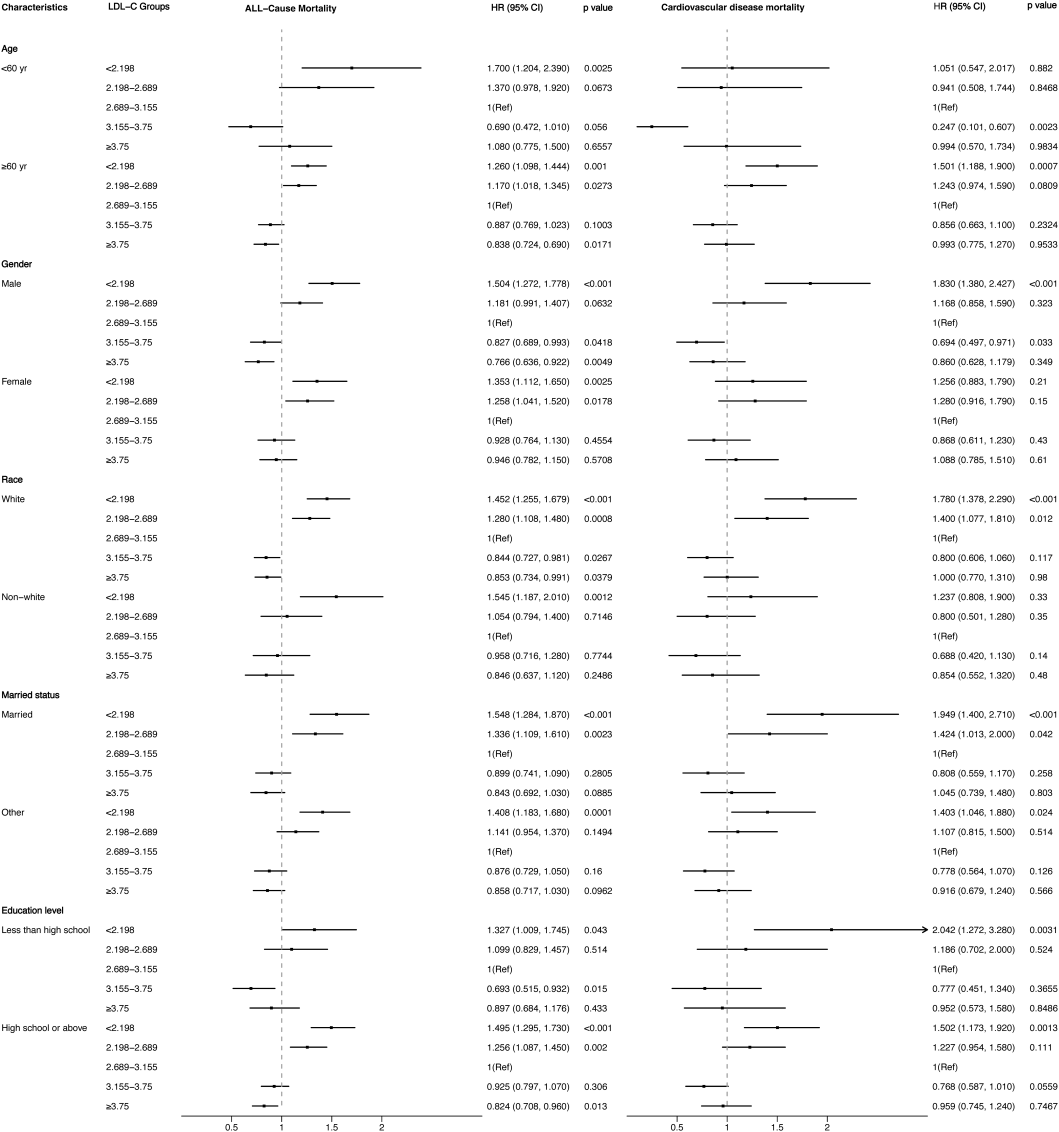
Stratified analysis of the relationship between LDL-C level and all-cause and cardiovascular mortality.

**Figure 7:**
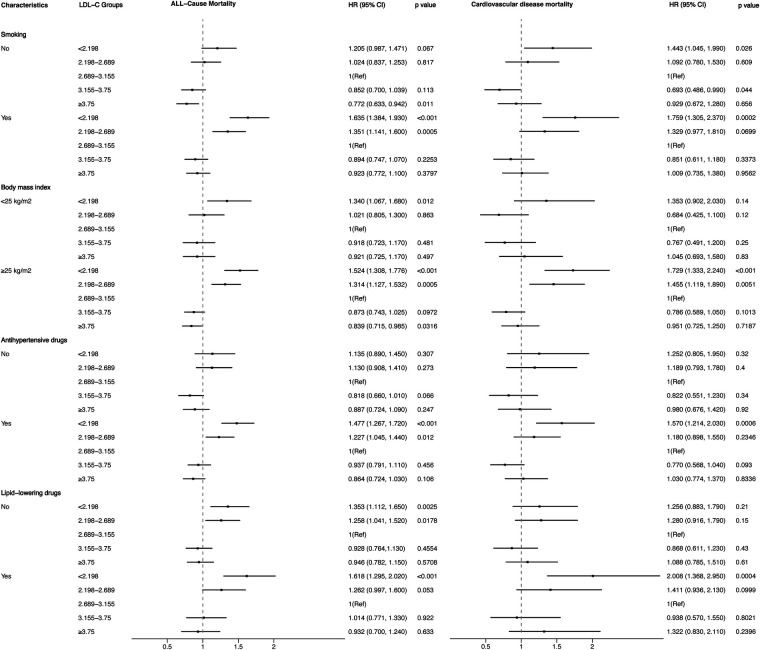
Stratified analysis of the relationship between LDL-C level and all-cause and cardiovascular mortality.

Figures 4, 5 showed the results of the Kaplan–Meier curve survival analysis. The curve was plotted by using LDL-C concentration as the independent variable, outcome variables as the occurrence of all-cause and cardiovascular deaths, and grouping by LDL-C level. As the results showed, both all-cause and cardiovascular mortality were significantly different from the other groups when LDL-C was at the lowest level. Figures 6, 7 presented the results of stratification analysis stratified by all confounders included in this study. Consistent with the results of univariate analysis, the lower level of LDL-C groups had a higher risk of all-cause and cardiovascular death in all subgroups. Specifically, we turned continuous variables age (<60 and ≥60 years) and body mass index (<25 and ≥25 kg/m^2^) into categorical variables for further study. As the results showed, patients aged <60 years with hypertension may have a higher risk of all-cause mortality when at a lower level of LDL-C. However, in the population aged ≥60 years, a higher risk of cardiovascular mortality was statistically significant. As to body mass index, both higher risk of all-cause and cardiovascular mortality were observed when body mass index ≥25 kg/m^2^. In particular, when subgroup analyses of antihypertensive and lipid-lowering medication use were conducted, they showed a higher risk of mortality in undertaking these two treatment measures [antihypertensive drugs: 1.477 (95% CI 1.267–1.720) in all-cause mortality, 1.570 (95% CI 1.214–2.030) in cardiovascular mortality; lipid-lowering drugs: 1.618 (95% CI 1.295–2.020) in all-cause mortality, 2.008 (95% CI 1.368–2.950) in cardiovascular mortality] than not among the lowest LDL-C level group. This result seems counterintuitive and less consistent with other results; it must be viewed and interpreted with caution. People taking these medications may already have comorbidities themselves instead of taking these drugs that cause increased mortality.

## 4 Discussion

In this retrospective cohort study of 9,635 hypertensive patients, our key finding was the non-linear association between LDL-C and all-cause and cardiovascular mortality. After adjusting for confounders of age, gender, race, marital status, level of education, smoking, body mass index, systolic and diastolic blood pressure, and medication use, the RCS curve based on multivariate-adjusted Cox regression well revealed the correlation between them on a continuous scale. Distinguishing from the traditional impression that lower LDL-C levels were better for health, we found both low and high LDL-C levels contributed to increased risk of death in the hypertension population. As to the lowest risk of all-cause and cardiovascular mortality, we had a preliminary view that the LDL-C concentration slightly higher than 2.89 mmol/L may be optimal in hypertensive patients according to the RCS curve. These new findings may provide some reference for lipid control in hypertensive populations.

As the major culprit in the development of atherosclerosis, there is no doubt that elevated levels of LDL-C are strongly associated with cardiovascular disease. According to statistics from the World Health Organization report in 2021, cardiovascular disease caused 17.9 million deaths in 2019, making up 32% of total global deaths (24). Therefore, it can be easily understood that higher levels of LDL-C are accompanied by a higher risk of death. However, when it comes to the lower LDL-C levels, the higher risk of death seems incomprehensible. For this counterintuitive result, there are several probable explanations. First, it is hypothesised that debilitation and disease can lead to lower cholesterol levels (17, 25, 26). In this study, patients with lower levels of LDL-C had an older age (Q1: mean age of 62.2 ± 16.0 years, Q2: mean age of 59.5 ± 16.2 years) than those with higher levels (Q4: mean age of 58.5 ± 15.4 years, Q5: mean age of 58.3 ± 14.6 years). Individual comorbidity profiles were not included in our study, but it can be inferred from individual medication histories that the low-level groups had higher percentages of medication use. Second, although most studies have spared no effort to emphasise the benefits of lipid-lowering, the long-term safety and efficacy of LDL-C lowering therapies remain a question to be further explored (27). Moreover, some studies have reported neurocognitive deficits, haemorrhagic stroke, and new-onset diabetes in the presence of reduced LDL-C (27–29), which may invariably increase the risk of all-cause mortality. Third, Kaysen et al. found that higher LDL-C was significantly associated with lower infection-related mortality in an international retrospective cohort study (13). In other words, the risk of infectious death may increase when LDL-C is at a low level and finally leading to increasing all-cause mortality. Finally, as the world's second most common cause of death, cancer was related to low LDL-C levels, which has been repeatedly mentioned in multiple studies (30–33). Therefore, reduced LDL-C levels might elevate the likelihood of mortality from the possible reason above, which then results in increased all-cause mortality.

Consistent with the results of our study, some previous studies conducted in other populations have demonstrated a correlation between LDL-C levels and the risk of all-cause and cardiovascular mortality. Zhou et al. and Tikhonoff et al. explored relationships in older people. Zhou et al. reported there was a U-shaped relationship between untreated LDL-C levels and all-cause mortality (34), and Tikhonoff et al. found that LDL-C concentration is a multifaceted risk factor in older adults (35). Chang et al. demonstrated that both lower and higher levels of mean LDL-C were associated with increased all-cause and cardiovascular mortality in patients with type 2 diabetes (36). In addition, multiple studies on the general population had similar results (17, 37–39). Further, through a prospective cohort study of 108,243 individuals in Denmark, Johannesen et al. found that the lowest risk of all-cause mortality was at LDL-C concentrations of 3.6–3.7 mmol/L (17). However, from the present study, lipid control in hypertensive populations should be even more strict.

### 4.1 Study strengths and limitations

Thanks to the ongoing NHANES project and continued data collection, we were able to build such a large sample size cohort of hypertensive people for our analysis. No individuals were lost to follow-up and the cause of death of every participant was recorded on the National Death Index death certificate records. As far as our knowledge extends, the relationship between low-density and all-cause and cardiovascular mortality remains controversial. A few studies explored it in general or in other populations; however, our study may be the first attempt to do explore it specifically in hypertensive populations. Another strength of our study is that we adjusted for several confounders, which may influence the accuracy of analysis results.

However, limitations should also be considered. First, the population we included was only living in the United States; other countries or ethnicities may not be applicable. Second, some of the variables we included, such as smoking status and medicine use, may cause recall bias because they were subjective from participants. Third, we did not consider changes in LDL-C concentration over time or changes influenced by the initiation or cessation of lipid-lowering treatment throughout the observation period, and this may make the findings unreliable. Finally, given the observational nature of the study, causality cannot be definitively established. Therefore, it is imperative to interpret the findings with caution, considering both potential causal and reverse relationships. Subsequent research is warranted to elucidate the possible causal link between LDL-C levels and mortality.

## 5 Conclusion

The present study revealed a non-linear association between LDL-C levels and both all-cause mortality and cardiovascular mortality in individuals with high blood pressure. Maintaining LDL-C within a specific range may confer benefits for cardiovascular health and long-term survival when compared to lower or higher concentrations. Nevertheless, additional research is necessary to determine the optimal LDL-C concentration range.

## Data Availability

The datasets presented in this study can be found in online repositories. The names of the repository/repositories and accession number(s) can be found in the article/Supplementary Material.
